# In vitro comparison of the effects of rough and polished stem surface finish on pressure generation in cemented hip arthroplasty

**DOI:** 10.3109/17453670902876755

**Published:** 2009-04-01

**Authors:** Gavin E Bartlett, David J Beard, David W Murray, Harinderjit S Gill

**Affiliations:** ^1^OOEC, Nuffield Department of Orthopaedic Surgery, University of OxfordOxfordUK; ^2^Nuffield Orthopaedic CentreOxfordUK

## Abstract

**Background and purpose** High pressures around implants can cause bone lysis and loosening. We investigated how pressures are generated around cemented femoral stems.

**Method** We compared the pressures generated by rough and polished tapered stems at their cement interfaces, in an in vitro model, before and after 1 million load cycles.

**Results** At the start of the study, the loading of both polished and rough stems generated interface pressures that were not statistically significantly different. After 1 million load cycles, the rough stems generated greater interface pressures than at the start (p = 0.03), with maximum pressure wave amplitudes of 450,000 Pa or 3,375 mm Hg. The pressures generated by polished stems were similar before and after 1 million load cycles, and were lower than the pressures generated by the rough stems (p = 0.01). Stem loading caused micromotion between the stem and cement. Polished stems migrated distally in the cement but retained rotational and axial stability. The rough stems also migrated distally and wore the cement mantle, leading to increased rotational instability.

**Interpretation** The change in the rotational micromotion of the rough stem is likely to be the principal cause of the increased stem pump output and to be a key factor in the longevity of cemented femoral implants.

## Introduction

Aseptic loosening of hip implants mainly occurs by osteolysis of periprosthetic bone, and contact with both particulate wear debris and pressurized fluid has been shown to stimulate this process (Harris 1994, Aspenberg and van der Vis 1998). However, contact with periprosthetic bone is limited by the cement mantle (Manley et al. 2002).

Debonded cemented femoral stems move within their cement mantles under physiological combinations of torsional and offset axial loads (Glyn-Jones et al. 2004). This stem movement has been shown in vitro to generate cyclical fluid pressure and flow at the stem-cement interface (Bartlett et al. 2008). In the presence of full thickness cement mantle defects, the femoral stem pump has the potential to facilitate loosening of cemented femoral stems by forcing high-pressure fluid and particles through the defects onto underlying bone (Bartlett et al. 2008). The surface finish of cemented stems has been found to influence fluid flow at the stem-cement interface under static conditions in vitro, and also to influence wear mechanisms at the stem-cement interface in studies of retrieved femoral stems (Crawford et al. 1999, Howell et al. 2004). Surface roughness may therefore have possible effects on the stem pump mechanism and offer an explanation for the different clinical performance of the polished (Stryker, Newbury, UK) and matt (Howmedica, Newbury, UK) Exeter stems. The polished stems have shown excellent results (Malchau et al. 2002, Williams et al. 2002); however, the matt Exeter stem with the same geometry but a rougher surface finish has been associated with a higher rate of distal femoral osteolysis not seen with the polished stems (Anthony et al. 1990, Malchau et al. 1993).

Our hypothesis in this study was that stems with a rough surface finish will generate higher fluid pressures at the stem-cement interface than those with polished surfaces. We also hypothesized that this could be due to 2 effects: initially by the rough surface producing less resistance to fluid flow at the debonded interface of a fully conforming mantle, and then over time causing stem loosening by abrasive cement wear—and thereby augmenting the stem pump mechanism.

## Method

**Figure 1.**
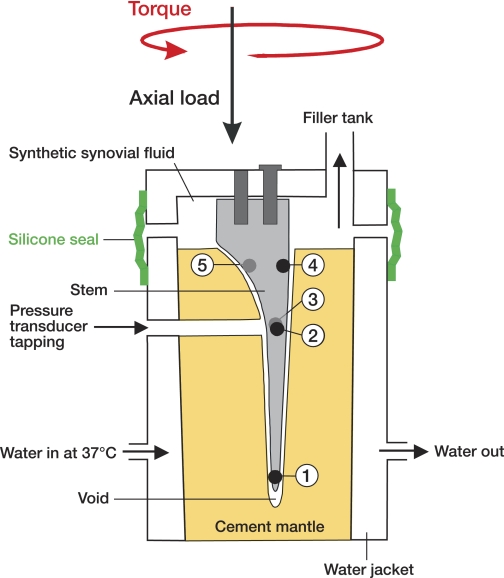
Cross-sectional view of HAPS apparatus with model stem in situ and showing sampling sites at the cement mantle-stem interface.

We assessed the in vitro performance of two groups of model Exeter type stems with either rough or polished surface finishes. Both groups were loaded physiologically for 1 million cycles and the interface pressures were measured at the start and at the end of the loading. The study was performed using the Hip Arthroplasty Pressure Simulator (HAPS) previously reported to be a reliable and reproducible in vitro model of stem pumping (Bartlett et al. 2008) (Figure 1).

The model stems were half-sized models of a 37.5 mm offset size 1 Exeter stem and manufactured from 316 stainless steel. The section of the stem proximal to the cement mantle was re-fashioned to allow it to be bolted to the lid of the HAPS chamber. The surface finishes of the stems were prepared by the manufacturer of the Exeter stem (Stryker Orthopaedics, Hérouville Saint Clair, France) and they were either polished to tolerances of the current Exeter stem (0.03 µm Ra) or shot-blasted to created surface roughness in excess of 2 µm Ra.

**Figure 2.**
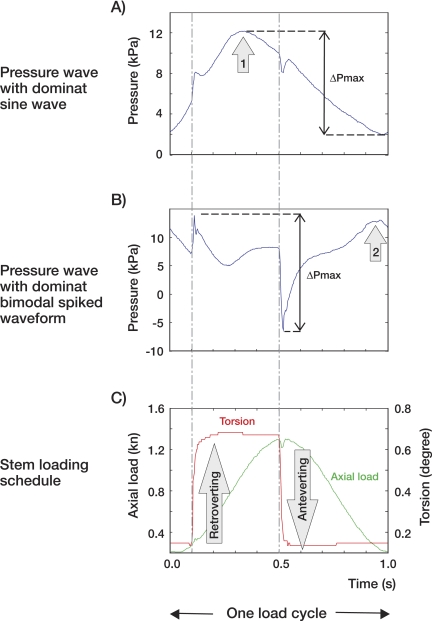
Typical pressure waves generated by a polished stem in a conforming cement mantle at 2 different sites, together with the stem loading schedule. The ΔPmax is the maximum pressure range developed over 1 load cycle (measured in pascals). The typical pressure wave is either dominated by a sine wave (A) or a bi-modal spiked waveform (B). The peaks of the sine wave (arrows 1 and 2) tend not to be synchronized with the peak of the applied axial load; however, the peaks in the bi-modal spiked waveform are synchronised with the torque loading.

The HAPS apparatus consists of a central testing chamber containing a cement mantle and a model stem, which were immersed in a synthetic synovial fluid (vegetable oil). The chamber and its contents were maintained at 37°C by circulation of heated water within the double-skinned outer wall and the resting pressure of the synovial fluid was maintained by a filler tank suspended over the apparatus. The pressure at the stem-cement interface was continuously sampled at 5 sites by transducers (PMP 1400; DRUCK Ltd, Leicester) situated on the exterior of the casing, communicating with the interface via pressure tappings that traversed the wall of the chamber and cement mantle. The tappings entered the interface at the following locations: the posteromedial (site 5) and anterolateral (site 4) aspects of the proximal mantle, the posterior (site 3) and anterior (site 2) surfaces of the mid-portion of the stem, and the implant tip (site 1). Thus, sampling sites 5 and 3 were on the posterior wall of the mantle, and 4 and 2 were on the anterior wall. These areas were predicted to experience the greatest flow rates based on the study of wear patterns on retrieved stems (Howell et al. 2004). The loads were applied to the lid of the HAPS chamber by a single-axis material testing machine (MTM) onto which an additional, horizontally mounted actuator had been added. The lid was bolted to the model stem and did not make contact with the chamber collar, therefore transmitting the loads directed to the stem. The loading schedule was derived from in vivo loads measured using instrumented prostheses implanted in subjects taking part in stair-climbing activities (Bergmann et al. 2001). The loading schedule used during pressure sampling was unchanged from that previously reported (Bartlett et al. 2008) with loads scaled to match the dimensions of the model stem. The axial, longitudinal forces were applied by the MTM actuator at the true medial offset of the model stem in a sinusoidal pattern oscillating between 1.3 kN and 0.2 kN, at a frequency of 1 Hz. Torsional forces were applied perpendicular to the longitudinal axis of the stem. These torsional forces were applied in a stepped pattern coordinated to the MTM actuator activity by the second actuator, coupled to the HAPS lid. The retroverting force of 8 Nm was greater than the anteverting force of 0.46 Nm, and occurred during the positive axial load ramp; this switched to the anteverting force during the negative axial load ramp (Figure 2C). The forces were increased by 150% during the 1 million loading cycles and the cycle rate was increased to 2 Hz. The 1 million cycles were performed in 100,000 cycle batches, which typically took 13 hours to complete. Each set of 100,000 load cycles was punctuated by a rest period of between 8 and 10 hours, to allow for stress relaxation of the cement. The increased magnitude and frequency of the loading schedule was designed to maximize the cement wear generated by the 1 million load cycles and reduce the time taken to complete the study, but maintaining the load schedules within the ranges experienced by prostheses in vivo (Bergmann et al. 1993).

3 mantles were assessed for each of the stem surface finishes; every mantle tested had 3 measurement trials performed at the start of loading (this time point was termed “Start”) and a further 3 were performed following the completion of 1 million load cycles. Each trial was punctuated by disassembly and reassembly of the apparatus and involved 500 initial load cycles to bed in the stem followed by a data sampling period of 30 load cycles. For each measured load cycle the magnitudes of the positive and negative pressure peaks were identified, as well as their timing within the cycle. The difference between the positive and negative peaks represents the maximum range of pressure recorded over the course of a single load cycle. This pressure range, termed ΔPmax and expressed in pascals, was used as the primary variable in this study (Figure 2).

### Statistics

All data were processed using custom-written Matlab routines (version 7; Mathworks, Inc., Natick, MA). The data were not normally distributed; therefore, a median ΔPmax value was used to represent the 3 trials sampled for each mantle at the start and finish of loading. As there were 3 mantles in each group, the mean value and standard deviation were used to describe the distribution of the 3 median ΔPmax values for each transducer site in the polished-finish and rough-finish study groups. A Wilcoxon signed ranks test was performed on the paired median ΔPmax values at the start and at the end of loading of all 5 transducer sites in the 3 mantles tested in the 2 study groups (n = 15 for each group). A Wilcoxon rank sum test was performed on the unpaired data from the polished-finish and rough-finish study groups at the start and at the end of loading (n = 15 in each group). All statistical analyses were performed using Matlab v.7 and statistical significance was set to α ≤ 0.05.

## Results

**Figure 3.**
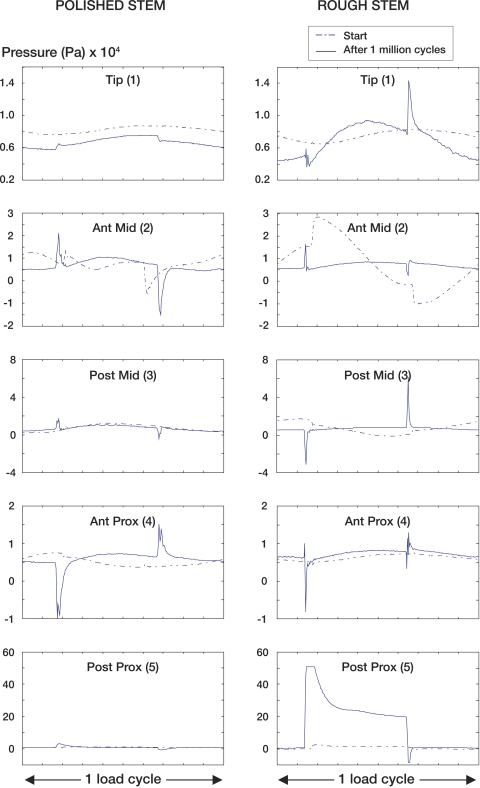
Comparison of the pressure waves of one load cycle generated by polished and rough stems taken at 5 sampling sites at the start of loading (dashed lines) and after 1 million load cycles (solid lines).

**Figure 4.**
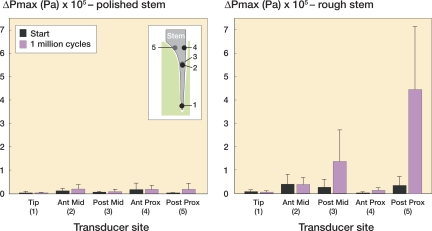
Average of median ΔPmax recorded at the start of the study and after completing 1 million cycles with both polished (A) and rough (B) stems (3 mantles were studied in each group).

The results are presented in 2 main forms: as illustrative examples of pressure waveforms generated during single load cycles (Figures 2 and 3) and as a summary of ΔPmax values generated by both stem surface finishes across all the mantles tested (Figure 4).

The pressure waveforms generated at the sampling sites at the start of the study by both stem types had 2 main components: a sinusoidal wave representative of the axial load schedule and a bi-modal spiked waveform due to the stepped changes in torsional loading. The overall pressure pattern observed at the individual sampling sites was a combination of these two waveforms, with either the sine wave dominating the overall behavior (Figure 2A) or the bi-modal spiked waveform dominating (Figure 2B). The recorded peak of the sine wave was often out of phase with that of the axial loading applied, and this phase shift varied with sampling site. However, the timings of the pressure spikes in the bi-modal spiked waveform were always synchronized to the torque load schedule (Figures 2 and 3).

At the start of loading, for both stem finishes the sine wave was dominant for the majority of the sampling sites (Figure 3). However, for both stem finishes, at sampling site 2—the anterior mid-point on the stem—the bi-modal waveform dominated the overall pattern (Figure 3). Overall, there was no statistically significant difference between the median values of ΔPmax developed by the rough and polished stems at the start of the study (p = 0.2). However, the highest average ΔPmax values recorded were generated by the rough stems at sites 2 and 5: 41,000 Pa and 35,000 Pa respectively (Figure 4). For both stem types, the peaks of pressure occurred at different time points in the load cycle at the various sample sites (Figure 3).

After 1 million cycles, there were marked changes in the pressure patterns observed. For both surface finishes at every sampling site, the bi-modal spiked waveform became more dominant; this was least evident at the tip (sample site 1) of the polished stem (Figure 3). The phasing of the bi-modal spiked waveform with the torque loading was either with a positive pressure spike during the retroverting torque followed by a negative pressure spike during the anteverting torque (termed bi-modal1) or with a negative pressure spike during the retroverting torque followed by a positive pressure spike during the anteverting torque (termed bi-modal2). For the polished stem, all sampling sites except site 4, the anterior proximal point on the stem, displayed bi-modal1 phasing. Sample site 4 showed bi-modal2 phasing. For the rough stem, sites 2 (the anterior mid-point) and 5 (the posterior proximal point) displayed bi-modal1 phasing; the other sites had bi-modal2 phasing (Figure 3).

For the polished stem finish, after loading there was no statistically significant change in the median ΔPmax values (p = 0.2) recorded at all 5 transducer sites (Figure 4). For the rough surface finish after loading, median ΔPmax values recorded at the 5 transducer sites increased compared to the start (p = 0.03); the ΔPmax values developed by the rough stems were also greater than the ΔPmax values recorded with the polished stems (p = 0.01) (Figure 4). The rise in rough stem interface pressures was not uniform across the 5 sampling sites: average ΔPmax values increased at 3 sites and remained similar at 2 (Figure 4). There was a 5-fold increase at site 3 (posterior mid-point), and a 4-fold increase at site 4 (anterior proximal point) and site 5 (posterior proximal point). ΔPmax was approximately 12 times greater than the starting value. The greatest changes were thus observed at the posterior proximal aspect of the stem, where the average values of ΔPmax were around 450,000 Pa (3,375 mmHg) after loading with a rough stem as compared to 35,000 Pa at the start (Figure 4).

After the loading there were changes to both the stem position within the cement and the recoverable micromotion of the stems during each load cycle. Both stem types migrated distally and into internal rotation; the polished stems migrated 0.4 mm distally with 0.5° internal rotation whereas the the rough stems migrated on average by 0.7 mm distally with 0.6° internal rotation. In association with this permanent displacement, the stems exhibited recoverable, oscillatory micromotion in response to each load cycle. The torsional micromovement of the polished stems increased over the loading cycles by an average of 0.01°, and that of the rough stems by 0.8° (Table). The increases in axial recoverable micromotion over the load cycles seen with either stem type were small in proportion to the migration distance; the axial micromotion of the polished surface finish stems increased by less than 0.01 mm, and that of the rough stems by 0.03 mm (Table).

**Table:** The increase in recoverable rotational and axial micromotion in polished and rough stems between the start of loading and when 1 million load cycles had been completed

Surface finish	Micromotion			
	Rotational (degrees)		Axial (mm)	
	Mean	SD	Mean	SD
Polished	0.01	0.08	0.005	0.009
Rough	0.81	0.41	0.028	0.030

SD: standard deviation.

## Discussion

The femoral stem pump mechanism has been shown to create oscillating pressure gradients and possible fluid flows at the debonded implant-cement interface of a polished double-tapered stem (Bartlett et al. 2008). Our hypothesis was that load cycling with rough surface finish stems would increase the pressures generated by the stem pump mechanism relative to those generated by load cycling with polished stems. This hypothesis is in agreement with the observations of Crawford et al. (1999) and Howell et al. (2004).

Within conforming mantles, i.e. at the start condition, the stems with rough surface finishes generated greater average ΔPmax values than polished stems, although there was no overall statistically significant difference, with mean ΔPmax values at 2 sampling sites (sites 2 and 5) being sufficient to induce macrophage differentiation without wear debris—i.e. 35,000 Pa or greater (McEvoy et al. 2002). This partially supports the findings of Crawford et al. (Crawford et al. 1999), that a rough surface finish offers less resistance to fluid flow than a polished surface finish at a conforming debonded interface. However, there was a statistically significant difference between ΔPmax values generated by stems with a rough surface finish and those generated by polished stems after 1 million load cycles. At this point, together with changes in the pressure waveform, there were significant increases in ΔPmax or the amplitude of the pressure waves generated by rough stems, especially along the posterior mantle wall.

Pressure gradients have been recorded at the interfaces of both stem types under physiological loads. The directions of gradients were seen to reverse during the course of each load cycle, providing evidence for possible oscillatory fluid flow at the stem-mantle interface, which is in keeping with results previously reported (Bartlett et al. 2008). The nature of the pressure gradients changed over the course of the experiment; at the start of loading, there appeared to be fluid flows distally to proximally on axial loading, and posteriorly to anteriorly as the stem was retroverted in the first half of the load cycle. These flows reversed in the second half of the load cycle. After the loading, the peak interface pressures generated by stems with either surface finish were coordinated to the torque load, with statistically significantly greater pressure gradients generated by the stems with a rough surface finish. For the rough surface finish, the trends in pressure measured indicated that fluid flows became more complex in the mid-to-proximal region of the stem, with the pressure generated in the posterior mantle wall increasing the most.

The increased pressures generated by the rough stems were linked to increased rotational micromotion of the stem compared to the polished stems, which remained rotationally stable. This was most likely due to a greater amount of cement wear generated by the rough surface finish. Both stems retained their axial stability. Interestingly, despite the increased micromovement of the rough stems, they remained well fixed in the cement, requiring substantial force to remove them. The increased rotational micromotion of the rough stems is consistent with wear patterns identified on explanted stems (Howell et al. 2004). Both stem types migrated into internal rotation by similar amounts, and therefore this movement did not distinguish the increased rotational stability of the polished stems compared to the rough. The migration of the stems may thus be secondary to different mechanisms of cement mantle wear such as creep. Both stem types were seen to migrate distally; this has been recorded previously in vivo using RSA techniques, and has been suggested to be a function of the dual taper geometry and hollow implant tip centralizer (Alfaro-Adrian et al. 1999).

In summary, under repetitive physiological loads both the rough and polished tapered stems migrated but the polished stems remained rotationally stable compared to the rough stems, which developed increased rotational instability.

The significant amplification of stem pump provides a potentially important mechanism for facilitatation of aseptic loosening of the stem by forcing fluid and wear debris through cement mantle defects and into direct contact with the underlying bone, causing lysis (McEvoy et al. 2002). This may have been the mechanism responsible for the higher failure rate seen with the matt Exeter stems (Howmedica) than with the polished ones (Stryker), and it offers an explanation for the reported high pressures measured within lytic cysts associated with cement mantle defects of failed matt Exeter stems (Anthony et al. 1990, Malchau et al. 1993). Furthermore, this may also have been the cause of increased failure rates for other cemented stems in which the surface finish was changed from polished to matt (Collis and Mohler 2002).

## References

[CIT0001] Alfaro-Adrian J, Gill HS, Murray DW (1999). Cement migration after THR. A comparison of Charnley Elite and Exeter femoral stems using RSA.. J Bone Joint Surg (Br).

[CIT0002] Anthony PP, Gie GA, Howie CR, Ling RS (1990). Localised endosteal bone lysis in relation to the femoral components of cemented total hip arthroplasties.. J Bone Joint Surg (Br).

[CIT0003] Aspenberg P, van der Vis H (1998). Fluid pressure may cause periprosthetic osteolysis. Particles are not the only thing.. Acta Orthop Scand.

[CIT0004] Bartlett GE, Beard DJ, Murray DW, Gill HS (2008). The femoral stem pump in cemented hip arthroplasty: An in vitro model.. Med Eng Phys.

[CIT0005] Bergmann G, Graichen F, Rohlmann A (1993). Hip contact forces and gait patterns from routine activities.. J Biomech.

[CIT0006] Bergmann G, Deuretzbacher G, Heller M, Graichen F, Rohlmann A, Strauss J, Duda GN (2001). Hip contact forces and gait patterns from routine activities.. J Biomech.

[CIT0007] Collis DK, Mohler CG (2002). Comparison of clinical outcomes in total hip arthroplasty using rough and polished cemented stems with essentially the same geometry.. J Bone Joint Surg (Am).

[CIT0008] Crawford RW, Evans M, Ling RS, Murray DW (1999). Fluid flow around model femoral components of differing surface finishes: in vitro investigations.. Acta Orthop Scand.

[CIT0009] Glyn-Jones S, Gill HS, Mclardy-Smith P, Murray DW (2004). Roentgen stereophotogrammetric analysis of the Birmingham hip resurfacing arthroplasty. A two-year study.. J Bone Joint Surg (Br).

[CIT0010] Harris WH (1994). Osteolysis and particle disease in hip replacement. A review.. Acta Orthop Scand.

[CIT0011] Howell JR, Blunt LA, Doyle C, Hooper RM, Lee AJ, Ling RS (2004). In vivo surface wear mechanisms of femoral components of cemented total hip arthroplasties: the influence of wear mechanism on clinical outcome.. J Arthroplasty.

[CIT0012] Malchau H, Herberts P, Ahnfelt L (1993). Follow-up of 92,675 operations performed 1978-1990.. Acta Orthop Scand.

[CIT0013] Malchau H, Herberts P, Eisler T, Garellick G, Soderman P (2002). The Swedish Total Hip Replacement Register.. J Bone Joint Surg (Am) (Suppl 2).

[CIT0014] Manley MT, D'Antonio JA, Capello WN, Edidin AA (2002). Osteolysis: a disease of access to fixation interfaces.. Clin Orthop.

[CIT0015] McEvoy A, Jeyam M, Ferrier G, Evans CE, Andrew JG (2002). Synergistic effect of particles and cyclic pressure on cytokine production in human monocyte/macrophages: proposed role in periprosthetic osteolysis.. Bone.

[CIT0016] Williams HD, Browne G, Gie GA, Ling RS, Timperley AJ, Wendover NA (2002). The Exeter universal cemented femoral component at 8 to 12 years. A study of the first 325 hips.. J Bone Joint Surg (Br).

